# Drug reaction with eosinophilia and systemic symptoms (DRESS): an unusual manifestation of multi-visceral abnormalities and long-term outcome

**DOI:** 10.15190/d.2023.9

**Published:** 2023-08-27

**Authors:** Kinal Paresh Bhatt, Fahed Alsoud, Adesh Prashad, Jose Ortega-Tola, Virendra Ravat Singh, Pooja Patel, George Michel

**Affiliations:** ^1^Larkin Community Hospital, South Miami, FL, USA

**Keywords:** DRESS, vancomycin, cefepime, antibiotic spacer, visceral involvement, corticosteroids.

## Abstract

Drug reaction with eosinophilia and systemic symptoms (DRESS), also known as drug induced hypersensitivity (DiHS) is a rare, however a severe hypersensitivity reaction with a mortality rate of up to 10%, accounting for 10 to 20% of all cutaneous drug reactions in hospitalized patients. The clinical features of DRESS/DiHS may be challenging to recognize and diagnose, since they are delayed, stepwise, and heterogeneous. The classic presentation of DRRSS/DiHS involves a combination of cutaneous, hematologic, and internal organ involvement with a 2 to 8 weeks latency between drug exposure and the onset of symptoms. Finding the culprit drug in our case was difficult as the patient was taking multiple antibiotics. Drugs such as vancomycin and cefepime used before the rash outbreak for post-reconstructive surgery for left toal knee arthroplasty (TKA) approximately four weeks before the onset of the rash are likely offending agents. This patient also had multi-visceral involvement with eosinophilia and systemic symptoms. The current treatment guidelines for DRESS/DiHS are primarily based on expert opinion, as no randomized control trials exist. After the prompt withdrawal of the offending drug, systemic corticosteroids seem to have shown the best outcome for patients. Delaying discontinuing offending medications and initiating corticosteroid treatment may lead to poor results. The present case emphasizes that the close observation of patients with drug eruption induced by antibiotics is imperative. Primary care team should be able to promptly diagnose patients with DRESS syndrome, detect causative drug, and play a crucial role in the timely evaluation and treatment to reduce mortality rate. The later phase disease relapse or autoimmune complications may occur up to 5 years following the initial presentation. Therefore, we advised the patient to have an outpatient follow up for appropriate testing, including but not limited to genetic susceptibility due to the high risk of relapse and emerging risk of autoimmune diseases.

## INTRODUCTION

Drug reaction with eosinophilia and systemic symptoms (DRESS), also known as drug-induced hypersensitivity reaction (DiHS), is a delayed-type IVb hypersensitivity reaction mediated by antiviral T cells^1,2^. In the past, DRESS/DiHS were referred to as “allopurinol hypersensitivity syndrome” and “anticonvulsant hypersensitivity syndrome.” This rare but severe hypersensitivity reaction has a mortality rate of up to 10%, accounting for 10 to 20% of all cutaneous drug reactions in hospitalized patients. The pathogenesis of DRESS/DiHS is multi-factorial, involving drug-exposure and genetic predisposition through specific human leukocyte antigen (HLA) alleles, metabolism defects, viral reactivation, and immune dysregulation. The clinical features of this syndrome may be challenging to recognize and diagnose as they are delayed, stepwise, and heterogeneous. The classic presentation of DRESS/DiHS involves a combination of cutaneous, hematologic, and internal organ involvement with a 2 to 8 weeks latency between drug exposure and the onset of symptoms^3,4^.

Peyriere et al. (2006)^5^ conducted a retrospective study of 216 cases of drug-induced cutaneous side effects with systemic symptoms between 1985 to 2000. The researchers discovered that DRESS/DiHS patients presented with polymorphic progressive cutaneous rash findings in nearly 100% of the cases, notably diffuse maculopapular inflammatory reactions that progressed to become more diffuse and redder (most common). Eosinophilia was the most common hematological abnormality in >50% of cases, followed by atypical lymphocytes, neutropenia, anemia, and thrombocytopenia. Lymphadenopathy was found in >80% of cases, more commonly in patients using minocycline^5^. The most involved internal organs are the liver (elevated liver enzymes as seen in hepatitis), kidney (acute kidney injury to severe interstitial nephritis), lungs (interstitial pneumonitis), heart (myocarditis, cardiogenic shock, chest pain, and tachycardia), and neurological involvement in up to third of all cases. Facial edema present in up to 75% of patients is correlated with the severity of the disease. It is generally hard to notice if the examiner is not familiar with the patient’s pre-existing appearance. Occasionally, erosion of the mouth and mucous membranes may be prominent and mistaken for Stevens-Johnson syndrome or toxic epidermal necrolysis (SJS/TEN), such as oral involvement^6^.

DRESS/DiHS can be differentiated into the early and late phases of the disease. The early phase of the disease is within the first two weeks of presentation. However, symptoms can relapse 2-4 weeks following the acute phase or upon weaning patients off steroid treatment^3,4^. While the mechanism of action is poorly understood, the longer duration, severity, and relapse of symptoms in the acute phase are associated with viral reactivation. Multiple studies are showing a connection between DRESS/DiHS and viral reactivation of human herpes virus-6 (HHV-6) (most common), HHV-7, cytomegalovirus (CMV), Epstein Barr virus (EBV), and herpes simplex virus (HSV). The diagnostic criteria established by the Japanese consensus group require evidence of HHV-6 infection to diagnose DiHS. However, the modified The Registry of Severe Cutaneous Adverse Reactions (RegiSCAR) validation criteria by Choudhary et al. (2021) evaluates hepatitis viruses A, B, and C (HAV/HBV/HCV)^7,8^. The later phase disease relapse or autoimmune complications may occur up to 5 years following the initial presentation. The time between the onset of DRESS/DiHS disease, removal of the suspected culprit drug, and resolution of symptoms is highly variable and dependent on the poorly understood host, culprit drug (e.g., half-life), and treatment factors^3,4^.

## CASE REPORT

**Figure 1:**
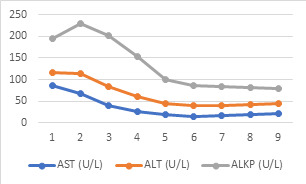
Trending liver enzymes

The medical team acquired informed consent from the patient to publish this case report. A 72-year-old male presented to the emergency department as a walk-in, complaining of a skin rash for two days. The rash was maculopapular at onset, started on his chest area, and spread to bilateral upper and lower extremities and back. He stated that at the beginning, the rash was moderately pruritic, complained of tongue swelling, and noted the rash worsened when he took a hot shower, inflicting burning pain. However, the patient denied fever, chest pain, shortness of breath, or palpitations. He also denied nausea, vomiting, or diarrhea. Following the primary left TKA (total knee arthroplasty) procedure, the patient was diagnosed with osteomyelitis. He underwent revision of the left TKA with antibiotic spacer placement about four weeks before this emergency room (ER) visit and took vancomycin and cefepime for approximately four weeks post-surgery. He stopped taking both antibiotics after the onset of the rash. The patient was also taking celecoxib and aspirin for pain and inflammation post-TKA, which were also discontinued upon the start of the rash. The patient had been taking niacin for several years for dyslipidemia. However, she/he did not take any other medications. The rash eruption was maculopapular on physical exam, without desquamation and exfoliation. After confirming he had no known drug or food allergies, the medical team advised the patient to stay off vancomycin, cefepime, aspirin, and celecoxib, as suspected culprits for drug-related rash. The patient was started on oral diphenhydramine for skin rash, switched to oral linezolid and levofloxacin antibiotics for post-TKA prophylaxis, and admitted under the internal medicine (IM) team’s care. The initial labs revealed low hemoglobin at 10 g/dl, and total leucocyte count was as expected. However, differential leucocyte count showed high eosinophils at 11.6%, lymphocytes at 20.6%, and neutrophils at 53.9%. Serum alanine aminotransferase (ALT) (117) and aspartate aminotransferase (AST) (86) and alkaline phosphatase (ALKP) (196), and blood glucose were high (144); however, the patient denied a history of diabetes or liver pathology (Table 1*, *Figure 1).

**Table 1:** Trending laboratory values throughout hospitalization

Labs	Day 0	Day 1	Day 2	Day 3	Day 4	Day 5	Day 6	Day 7	Day 8
WBC (10^3/UL)	5.79	6.89	10.37	19.17	16.3	11.06	11.1	10.5	9.75
HGB (g/dL)	10	10.5	12.2	13.5	11.7	9	8.5	8.5	8.7
Iron (mcg/dL)	36	-	-	-	-	-	-	-	-
PLT (10^3/UL)	172	170	182	248	233	217	235	253	235
%NEUT	53.9	50.9	46.1	52.5	59.4	58.6	52.8	47.8	49.1
%LYMPH	20.6	27.1	32.1	19.6	24.7	32.5	37.4	41.4	34.3
%EOS	11.6	10.4	10.7	10.5	3.3	2.3	1.3	0.3	1.3
#NEUT	3.12	3.5	4.79	10.04	9.7	6.5	5.84	3.12	3.12
#LYMPH	1.19	1.87	3.33	3.76	4.03	3.59	4.14	1.09	1.09
#EOS	0.67	0.72	1.11	2.02	0.54	0.25	0.14	0.13	0.09
BUN (mg/dL)	19	18	17	25	34	34	38	36	36
Creatinine (mg/dL)	1.11	1.11	1.16	1.53	1.24	1.07	1.27	1.08	1.19
GFR (ml/min/1.73)	69	69	66	48	61	72	59	71	64
AST (U/L)	86	68	39	26	19	15	18	20	21
ALT (U/L)	117	115	85	61	45	39	41	42	44
ALKP (U/L)	196	229	201	153	100	87	85	81	79
TSH (mIU/ml)	-	-	-	-	3.35	-	1.22	-	-
Free T3 (pmol/L)	-	-	-	-	2.73	-	2.13	-	-
Free T4 (pmol/L)	-	-	-	-	0.65	-	0.57	-	-
Glucose (mg/dL)	144	112	130	171	191	180	201	153	112
Troponin (ng/mL)	-	-	-	0.012	-	-	-	-	-
CK (U/L)	-	-	-	<20	-	-	-	-	-
CMV IGG (U/mL)	-	-	-	-	-	-	-	IgG+	-
CMV IGM (U/mL)	-	-	-	-	-	-	-	IgM+	-
EBV IGG (U/mL)	-	-	-	-	-	-	-	IgG+	-
EBV IGM (U/mL)	-	-	-	-	-	-	-	IgM+	-
CRP (mg/dL)	-	-	-	-	-	-	-	0.9	-
ESR	25	-	-	-	-	-	-	9	-
LDHI	-	-	416	-	-	-	-	-	-
Lactic Acid	-	-	-	4.10	3.1	2.50	1.1	-	-
RPR	-	-	-	Negative	-	-	-	-	-

**Figure 2:**
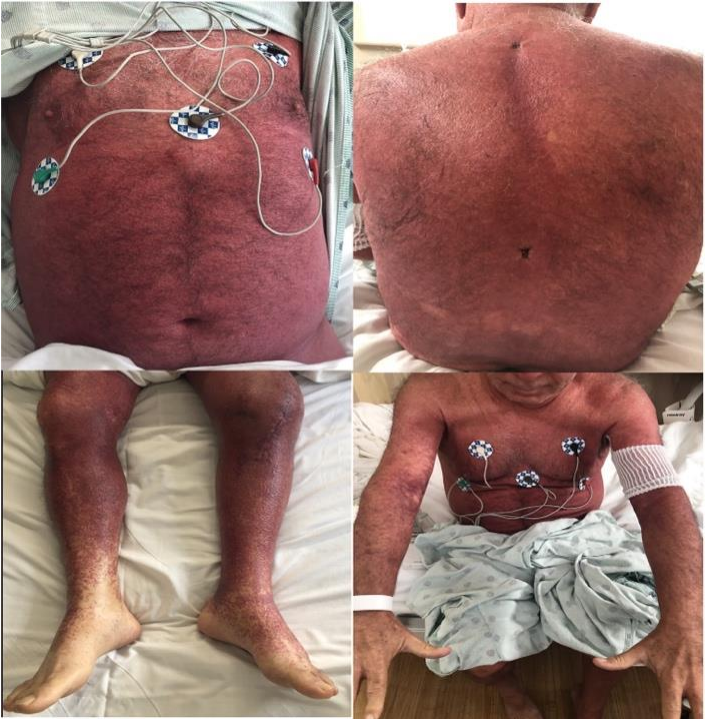
Rash of the patient, 3 days after onset By day 3, the rash continued to worsen, spreading more diffusely across the affected areas initially and now applying to palms and soles, further darkening of the rash was also seen.

By day 3, the rash continued to worsen, spreading more diffusely across the affected areas initially and now applying to palms and soles, further darkening the rash (Figure 2). The patient had reported only mild relief from pruritis; topical triamcinolone seemed to help relieve symptoms. The patient was experiencing nausea, likely secondary to antibiotics.

Ondansetron seemed to help relieve symptoms. By day 4, the patient reported new onset occipital headache and blurry vision, with no other associated symptoms. The medical team also recorded moderate right axillary and mild left occipital lymph node swelling. Antibiotics linezolid and levofloxacin were discontinued for presumed offending agents worsening the symptoms, and the patient was switched to intravenous aztreonam and daptomycin for TKA infection prophylaxis. The dermatology team obtained three punch biopsies of the patient’s skin as the medical team suspected DRESS/DiHS, which pathologists confirmed to be with perivascular eosinophils on histology, confirming the diagnosis of DRESS/DiHS. The patient’s RegiSCAR score was recorded at 6.5, an approved diagnostic method to identify DRESS. By day 3, the patient’s renal function had also declined (blood urea nitrogen (BUN) (29)/creatinine (1.53)), he developed bilateral lower extremities edema (2+), complained of burning when swallowing (denied dysphagia or difficulty swallowing solids or liquids), developed facial swelling, and redness, AST, ALT, ALKP, and glucose level continued to trend high. The floor nurse had called code sepsis for tachycardia (heart rate of 111) and leukocytosis. At this time, all antibiotics were discontinued, and the patient was started on IV corticosteroids. He was transferred to the intensive care unit (ICU) for close observation.

On days 4 and 5, the patient showed resolution of eosinophilia, liver dysfunction, and progressive improvement in skin rash within 24 hours after treatment with systemic corticosteroids. His facial swelling and redness, bilateral lower extremity edema, and symptoms of burning while swallowing had resolved. He reported complete relief from pruritis within 24 hours of topical triamcinolone application on surface areas affected by the rash. On day 4, the patient was downgraded from the ICU floor. However, on day 5 of the hospitalization, the patient’s viral panel came back positive for Cytomegalovirus (CMV) (IgM and IgG) and Epstein Barr Virus (EBV) (IgM and IgG), indicating high-risk viral reactivation and associated risk for a longer duration, severity, and risk of relapse of symptoms after weaning off steroid treatment. The medical team continued to taper the IV corticosteroid dose to oral prednisone, ensuring the patient remained clinically and hemodynamically stable. The patient had declined past medical history of thyroid disorders; however, his labs confirmed low triiodothyronine (T3) and thyroxine (T4) hormones on day 4, indicating hypothyroidism secondary to DRESS/DiHS. On days 7 and 8, the patient remained stable, and the rash had improved as spots of original skin color could be seen. The medical team thoroughly educated him on the importance of maintaining his outpatient follow-ups with a primary care physician, allergy and immunology specialist, and endocrinologist for follow-up labs and diagnostic tests.

## DISCUSSION

Due to high mortality in up to 10% of cases, DRESS/DiHS is considered a dermatological emergency^9^. While the exact pathogenesis of DRESS/DiHS is not entirely understood, proposed contributing factors include an exposure to an extensive list of drugs, genetic predisposition through HLA alleles, metabolic and viral reactivation, notably HHV, such as HHV-6 and -7, EBV, CMV, and hepatitis A, B, C^3,10,11^. Immunologic reaction with possible viral involvement associated with decreasing circulating B cells and serum immunoglobulin levels, which causes the viral reactivations leading to a more severe systemic immune response. Inflammatory cytokinesm, such as the Interleukin 5, have been reported to contribute to organ injury and promote eosinophilia^10^. The patient, in this case, was taking Niacin as an anti-hyperlipidemia drug. There was no rash or fever during the patient’s long-term use of oral anti-hyperlipidemia drugs. Therefore, this drug was less likely to be the etiology of DRESS/DiHS in this patient. The possible offending drug was used before the rash outbreak, which points to vancomycin and cefepime added post-reconstructive surgery for left TKA approximately four weeks before the onset of the rash. The patient's only clinical symptoms at onset were a diffuse maculopapular rash with swelling of the tongue that appeared 30 days after starting these medications, latency consistent with that reported by Kardaun et al.^12^.

Finding the culprit drug in our case was difficult as the patient took multiple antibiotics. vancomycin in our patient was a highly suspicious culprit after reviewing that the patient underwent revision of the left TKA with antibiotic spacer placement. The national incidence of peripheral joint infection (PJI) is relatively low (2.4%) after TKA; however, it is still one of the most devastating complications if not diagnosed and eradicated on time to prevent re-implantation prevent recurrence of the infection. While the antibiotic spacer cement maintains joint space and stability, it also protects the joint from infection by directly providing high concentrations of antibiotics to the infected bone; such precision is more difficult with systemic antibiotics^13^. Vancomycin is established for treating various infections due to the increasing prevalence of methicillin-resistant staphylococcal aureus (MRSA). The literature search identified 3 cases of vancomycin-induced DRESS/DiHS in patients with prosthetic joint surgeries^14,15^. Since vancomycin and cefepime were suspected of DRESS/DiHS, the patient was discontinued on both antibiotics and switched to linezolid and levaquin at admission. On Day 2, due to the worsening of the rash and new onset nausea, the patient was again changed to aztreonam and daptomycin. The Orthopedics surgery team was also consulted and determined that the patient’s antibiotic spacer was intact, there were no signs of infection in the operated joint, and no acute intervention was needed. They decided vancomycin was less likely to be the source of DRESS/DiHS due to its low incidence (2 to 5% of all cases). Therefore, cefepime and other drugs of the same class were determined to be a highly suspicious culprit of the patient’s symptoms^16^. The spacer containing gentamicin and vancomycin are presumed to elute a negligible amount of antibiotics at approximately ten days post-implantation, 0.05% to 0.4% and 0.8% to 3.3%, respectively. The patient’s knee did not have any signs of infection on clinical exam or imaging; therefore, removing the spacer at this time would offer no benefit to the patient^15,17-20^.

Although there are a variety of etiologies, drugs are the most common cause of severe skin diseases. The heterogeneous clinical presentation of DRESS/DiHS makes it a challenging diagnosis. Other more commonly misdiagnosed severe cutaneous adverse drug reactions (SCARs) such as Stevens-Johnson syndrome (SJS), toxic epidermal necrolysis (TEN), acute generalized exanthematous pustulosis (AGEP), and erythroderma were ruled out by working with our multidisciplinary team consisting of the dermatologist, infectious diseases, allergy and immunology specialists. The patient manifested diffuse maculopapular exanthema (MPE), which was urticarial, eczematous, significant dermal edema and inflammation, and had pustular manifestation (Figure 1). Widespread erythroderma persisted even after the potentially causative drugs were withdrawn. Edema and erythema on the face are highly characteristic of DRESS/DiHS and indicate the worst prognosis in these patients, which were present in our patient. DRESS/DiHS almost always starts with a high fever, which was absent in this patient and could have easily offset the accuracy of the diagnosis. The onset of SJS, TEN, AGEP, erythroderma, and many other drug-related SCARs can be anywhere from within 48 hours to less than three weeks of drug exposure. Lack of bullae, atypical target lesions, positive Nikolsky's sign, and mucocutaneous erosions ruled out the possibility of SJS/TEN. SJS, TEN, and AGEP also have a shorter duration of an eruption of approximately <1 week to up to 3 weeks. This patient had lymph node enlargement in the right axillary and left occipital nodes, unique to DRESS/DiHS. Pathology report obtained by obtaining skin biopsy showed spongiotic dermatitis with perivascular eosinophils, confirmed diagnosis of DRESS/DiHS. SJS/TEN, AGEP, and erythroderma, respectively, would show epidermal necrosis, subcorneal pustules, and lymphoma on a histological pattern of the skin^3,21,22^.

Peripheral blood counts, liver function tests, urinalyses, and pathogenic detection to excluding autoimmune or infectious diseases as the cause of DRESS/DiHS were performed. Cacoub et al. (2011)^16^ showed probable and definite cases of DRESS/DiHS demonstrate the delayed onset of symptoms compared to possible cases. This is consistent with our patient, who exhibited a long latency period of 4 weeks and scored 6.5 points on RegiSCAR, which indicates a "definite" DRESS case^16^. DRESS/DiHS is a diagnosis of exclusion as there are no pathognomonic signs or diagnostic tests for it. The gold standard to diagnose drug eruption is re-challenging the patient with the causative drug; however, this can be life-threatening. Our patient was unaware if he had been exposed to any antibiotics given to him throughout his hospitalization or the recent antibiotics prescribed post-TKA. Depending on the specific organ involvement, the differential diagnosis may be explored; viral hepatitis (liver), glomerulonephritis, vasculitis, pre- and post-renal causes of acute kidney injury (kidney), Kawasaki and eosinophilic myocarditis (heart), parasitic infection (gastrointestinal tract), and bacterial, viral, and fungal pathogens (lung). The extent and severity of organ involvement are directly correlated to the severity of DRESS/DiHS^23^.

This patient had elevated transaminases. However, no hepatomegaly. A study of 25 patients by Lee et al. (2017)^24^ and 60 patients from Taiwan by Chen et al. (2010)^9^ demonstrated liver involvement in 80% of the cases. Liver enzymes trended down within 24 hours of the patient's start on IV corticosteroids. The study by Lee et al. (2017) also showed renal involvement in 11%-28% of patients manifested as elevation in creatinine, decrease in glomerular filtration rate (GFR), proteinuria, and hematuria. Our patient exhibited 2+ proteinuria, elevation of BUN, and intermittent elevation of creatinine, while his GFR remained normal throughout the course of the hospitalization. Normal saline was added as temporary renal replacement therapy with resolution of proteinuria and return of creatinine to pre-morbid levels with the maintenance of GFR. Although lungs and cardiovascular involvement are rare in DRESS syndrome, they are associated with a high mortality rate. Some common findings are myocarditis, interstitial pneumonitis, pneumonia, pleural effusion, and acute respiratory distress syndrome (ARDS). Our patient had three chest X-rays throughout his hospitalization for close monitoring, all with unremarkable findings. Myocarditis can occur up to four months after all symptoms have resolved, all laboratory values have normalized, and the patient had successful treatment of DRESS. Our patient had at least one episode of tachycardia (Heart rate of 111) during his hospitalization. He was appropriately monitored on telemetry with an electrocardiogram (ECG), accompanied by unremarkable creatine kinase-MB (CK-MB) and troponin values. The patient had no significant ECG changes^9,16,24-26^.

There is a pathogenic connection between drug-specific immune response, which induces virus reactivation of human herpesvirus (HHV), herpes simplex virus (HSV), Epstein Barr virus (EBV), cytomegalovirus (CMV), and hepatitis viruses during the acute phase of DRESS syndrome. Viral reactivation typically occurs 2-4 weeks after symptoms onset and has been associated with longer duration, increased relapses, and severe outcomes of DRESS (lymphadenopathy, hepatitis, renal failure, encephalitis, myocarditis, severe lymphopenia, and death). This patient had serologic antibody screening tests for virus re-infection: CMV and EBV IgM and IgG antibody were positive. The serology for syphilis in this patient was negative^11,27-31^. A systematic review by Picard et al. (2010)^27^ found viral reactivation in HHV in more than 80% of cases. Another systematic review by Picard et al. (2010)^27^ found viral reactivation with a combination of EBV and HHV in 76% of cases. On Day 3, the patient reported new onset occipital headache, facial swelling, redness, burning sensation when swallowing (no dysphagia), bilateral edema of lower extremities, and right axillary and left occipital lymphadenopathy were palpated. However, the patient denied fever, chills, malaise, muscle weakness, confusion, disorientation, nausea or vomiting, delirium or hallucination, light sensitivity, stiff neck, and reduced touch sensation. The patient remained alert and oriented to person, place, and time. At this time, all antibiotics were discontinued as suspected contributing factors to DRESS. Computed Tomography (CT) of the brain without contrast confirmed prepontine mass, likely to be a meningioma. The patient confirmed he was diagnosed with prepontine meningioma approximately 17 years ago, and the lesion has remained stable in size without any new or worsening symptoms.

The emergence of autoimmune disease in DIHS/DRESS is poorly understood, and genetic susceptibility is thought to be a contributing factor. Genetic susceptibility may contribute to their development. According to an analysis of 34 cases by Kano and Shiohara (2013)^31^ of DiHS/DRESS, autoantibodies such as an anti-nuclear antibody (ANA), anti-thyroperoxidase (TPO), and anti-thyroglobulin (TG) antibodies are observed in patients during observational phase as well as after clinical resolution. The incidence of autoantibodies is higher in DRESS/DiHS patients than in SJS/TEN patients, particularly in patients not treated with systemic corticosteroids. This patient denied a history of thyroid-related diagnosis. However, they had low T3 and T4 hormones without any hypothyroid-associated symptoms. Some cases have also reported fulminant type 1 diabetes mellitus (FT1DM) associated with DRESS/DiHS, characterized by abrupt onset of high glucose, absence of islet-related autoantibodies, and destruction of pancreatic beta-cells. Patients may present with symptoms of vomiting and epigastric pain. Laboratory examination will have hyperglycemia, hyperosmolarity, and metabolic acidosis. Our patient denied a history of diabetes; however, they presented with hyperglycemia, hyperosmolality, and lactic acidosis during their hospitalization. Patients with FT1D may have an onset of elevated pancreatic enzymes; however, this patient was not tested for amylase and lipase. The onset of autoimmune diseases during or after DRESS/DiHS might get overlooked without the long-term follow-up of patients^31,32^.

The current treatment guidelines for DRESS/DiHS are primarily based on expert opinion, as there have been no randomized control trials. After the prompt withdrawal of the offending drug, systemic corticosteroids have shown the best outcome for patients. Delaying discontinuing offending medications and initiating corticosteroid treatment may lead to poor results. Cyclosporine, intravenous immunoglobulin (IVIG), plasmapheresis, or cyclophosphamide may be used without responding to corticosteroids. Antiviral drugs such as IV ganciclovir or oral valganciclovir are also suggested in patients with severe complications such as encephalitis, hemophagocytosis, or severe colitis when viral reactivation is present^7^. This patient was frequently re-assessed alongside the initiation of treatment with corticosteroids. Therapy with IV corticosteroids helped resolve this patient's transaminitis and eosinophilia by day 4 and all other symptoms except the full resolution of skin rash by day 5; however, the rash had started to improve by day 5. The relapse or autoimmune complications of DRESS/DiHS may occur up to five years after the initial presentation^6^. Due to the high risk of relapse, and emerging risk of autoimmune diseases, the patient was advised to follow up outpatient for appropriate testing, including but not limited to antinuclear antibody (ANA), anti-thyroid peroxidase antibody (anti-TPO Ab), anti-thyroglobulin antibody (anti-TG Ab), thyroid ultrasound, and genetic susceptibility.

## CONCLUSION

Despite significant advances in understanding the pathophysiology of DRESS/DiHS, it remains a challenging diagnosis due to its delayed onset, stepwise evolution, and heterogeneous clinical features. There is often an asymptomatic interval after the resolution of the acute disease. The relapse or autoimmune complications of DRESS/DiHS may occur up to five years after the initial presentation. Due to the high risk of relapse, and emerging risk of autoimmune diseases, the patients should be advised to follow up outpatient for appropriate testing, including but not limited to genetic susceptibility. The current treatment guidelines for DRESS/DiHS are primarily based on expert opinion, as no randomized control trials exist. After the prompt withdrawal of the offending drug, systemic corticosteroids seem to have shown the best outcome for patients. Delaying discontinuing offending medications and initiating corticosteroid treatment may lead to poor prognosis. The present case emphasizes that the close observation of patients with drug eruption induced by antibiotics is imperative. Primary care team should be able to promptly diagnose patients with DRESS syndrome, detect causative drug, and play a crucial role in the timely evaluation and treatment to reduce mortality rate. The later phase disease relapse or autoimmune complications may occur up to 5 years following the initial presentation. Therefore, we advised the patient to have an outpatient follow up for appropriate testing, including but not limited to genetic susceptibility due to the high risk of relapse and emerging risk of autoimmune diseases.
